# Trimming of sequence reads alters RNA-Seq gene expression estimates

**DOI:** 10.1186/s12859-016-0956-2

**Published:** 2016-02-25

**Authors:** Claire R. Williams, Alyssa Baccarella, Jay Z. Parrish, Charles C. Kim

**Affiliations:** Department of Biology, University of Washington, Seattle, WA 98195 USA; Division of Experimental Medicine, Department of Medicine, University of California San Francisco, San Francisco, CA 94110 USA; Present address: Verily, Mountain View, CA 94043 USA

**Keywords:** RNA-Seq, Trimming, Gene expression, *Drosophila*

## Abstract

**Background:**

High-throughput RNA-Sequencing (RNA-Seq) has become the preferred technique for studying gene expression differences between biological samples and for discovering novel isoforms, though the techniques to analyze the resulting data are still immature. One pre-processing step that is widely but heterogeneously applied is trimming, in which low quality bases, identified by the probability that they are called incorrectly, are removed. However, the impact of trimming on subsequent alignment to a genome could influence downstream analyses including gene expression estimation; we hypothesized that this might occur in an inconsistent manner across different genes, resulting in differential bias.

**Results:**

To assess the effects of trimming on gene expression, we generated RNA-Seq data sets from four samples of larval *Drosophila melanogaster* sensory neurons, and used three trimming algorithms—SolexaQA, Trimmomatic, and ConDeTri—to perform quality-based trimming across a wide range of stringencies. After aligning the reads to the *D. melanogaster* genome with TopHat2, we used Cuffdiff2 to compare the original, untrimmed gene expression estimates to those following trimming. With the most aggressive trimming parameters, over ten percent of genes had significant changes in their estimated expression levels. This trend was seen with two additional RNA-Seq data sets and with alternative differential expression analysis pipelines. We found that the majority of the expression changes could be mitigated by imposing a minimum length filter following trimming, suggesting that the differential gene expression was primarily being driven by spurious mapping of short reads. Slight differences with the untrimmed data set remained after length filtering, which were associated with genes with low exon numbers and high GC content. Finally, an analysis of paired RNA-seq/microarray data sets suggests that no or modest trimming results in the most biologically accurate gene expression estimates.

**Conclusions:**

We find that aggressive quality-based trimming has a large impact on the apparent makeup of RNA-Seq-based gene expression estimates, and that short reads can have a particularly strong impact. We conclude that implementation of trimming in RNA-Seq analysis workflows warrants caution, and if used, should be used in conjunction with a minimum read length filter to minimize the introduction of unpredictable changes in expression estimates.

**Electronic supplementary material:**

The online version of this article (doi:10.1186/s12859-016-0956-2) contains supplementary material, which is available to authorized users.

## Background

Within the past decade, RNA sequencing (RNA-Seq) has supplanted microarrays as the preferred technique for gene expression analysis. A typical workflow for RNA-Seq analysis involves aligning reads to an annotated genome followed by estimation of gene-level and/or isoform-level expression. In many cases, this is followed by statistical identification of genes that are differentially expressed between two or more sample groups. However, RNA-Seq presents unique analytical challenges, and accurate and robust tools to analyze sequencing data are rapidly evolving. As a result, analysis workflows can vary widely between studies.

One initial step of RNA-Seq analysis is to evaluate sequence read quality, which can vary substantially based on factors related to nucleic acid library preparation (*e.g.*, adapter contamination, polymerase errors) and sequencing (*e.g.*, cluster density, optical detection errors, phasing errors) [1]. For example, during library preparation, random hexamers are sometimes used as primers for double stranded cDNA synthesis, which leads to biases in nucleotide composition at the beginning of reads [2]. A second, intrinsic problem of sequencing by synthesis is phasing: different fragments within a cluster fall out of phase with one another as a result of slight differences in the timing of polymerization. Errors in phasing accumulate over time; thus, read quality tends to decrease toward the ends of sequence reads. Further, errors have a tendency to co-occur, such that reads with two errors are more common than would be predicted based on a model in which errors occur independently of one another [3].

In the absence of pre-processing, phasing and other sequencing errors can lead to inclusion of incorrect base calls and, consequently, to erroneous read alignment. Current next generation sequencing technologies produce reads as short as 25 bases up to hundreds of bases; sequencing errors are less frequent in the shorter read data sets, but the proportional impact of a single incorrect base may be larger. Sequencing-associated errors are aggregated into a quality score that reflects the probability that a given base has been called incorrectly. Most common among these, the Phred quality score (Q) used in the Illumina platforms ranges from 0 to 40, with increasing scores corresponding to higher quality base calls; for example, a Q score of 40 represents a 1 in 10,000 chance that a base has been called incorrectly [4]. Similar quality scores are produced with alternative sequencing platforms as well. During pre-processing, the quality score can be used to eliminate poor quality bases that typically occur at the ends of reads, in a procedure commonly referred to as “trimming”. This quality-based trimming is distinct from adapter trimming, which can be used to remove high quality internal bases matching the sequencing adapters used in library preparation [5]. Numerous approaches to quality-based trimming exist [6], all with the end result of retaining high quality internal bases while removing lower quality flanking bases.

However, as for pre-processing in general, quality-based trimming of reads is widely, but heterogeneously, applied. Thus, the specific algorithms and parameters used for quality score-driven trimming are a major determinant of what portions of reads are retained for further analysis. A broad survey of the major trimming algorithms currently in use found that although trimming prior to mapping of RNA-Seq reads leads to a decrease in the total number of reads, it concurrently increases the proportion of the remaining reads that map, suggesting that trimming is effective in removing reads that could not be mapped to the reference genome [6].

Although the above study suggested that trimming is beneficial, multiple lines of evidence suggest that it can also have detrimental effects. First, while errors in the assembly of a known transcriptome decrease with increased trimming, there is a concomitant decrease in the number of matching paired reads mapped, as well as the number of ORFs that can be identified [7]. Second, the number of distinct transcripts detected through *de novo* assembly decreases with more stringent trimming [8]. Finally, trimming can increase the number of false positive variant calls in genome sequencing studies [9]. All of these findings are consistent with increasing difficulty in unambiguously aligning shorter reads to a reference genome and/or reconstructing less total sequence into longer contiguous sequences.

The above studies have all investigated the influence of trimming on the immediately downstream steps of read alignment and transcriptome reconstruction [6–9], but it remains to be determined how trimming impacts further downstream analyses – for example, expression estimation and statistical identification of differentially expressed genes. One might expect that the specificity of read alignments could impact gene expression estimates and have vital effects on differential expression predictions. Consistent with this possibility, removing the first ten bases from all reads, irrespective of quality scores, led to an approximately two percent decrease in the number of differentially expressed genes detected in the *D. melanogaster* larval central nervous system following neuronal knockdown of a factor involved in spliceosome assembly [10]. More generally, one might expect that aggressive quality-based trimming would decrease the likelihood of detecting false positives that arise from erroneous mapping due to sequencing errors, while simultaneously reducing the sensitivity of detecting differentially expressed genes, since expression estimates would have reduced precision as a consequence of less sequencing information contributing to their measurement.

Here, we set out to explore the effects of quality-based trimming on gene expression analysis and report that multiple forms of bias in gene and isoform expression levels are apparent when comparing an untrimmed RNA-Seq data set to the same data set with trimming applied. Most of this bias can be removed by imposing a minimum read length requirement following trimming, suggesting that the gains in base calling accuracy that result from aggressive trimming are offset by the detrimental effects of estimating gene expression from short reads. However, despite the ability to correct much of the short read-associated bias by imposing a minimum length filter, a subset of biased genes remains resistant to correction. Thus, we caution that aggressive trimming of RNA-Seq data can introduce bias and unpredictability into RNA-Seq gene expression estimates, which can subsequently impact downstream differential expression analysis.

## Results and discussion

### Quality-based trimming of ultralow-input RNA-Seq data increases mappability

**Fig. 1:**
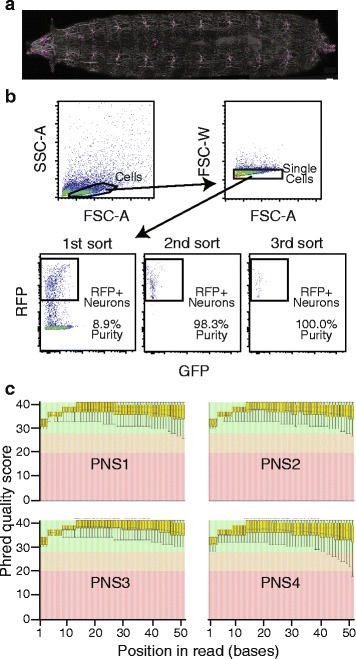
High quality RNA-Seq data generated from *D. melanogaster* sensory neurons. **a** Confocal image of *Drosophila* larval sensory neurons expressing a nuclear-targeted version of mRFP (magenta) and a membrane-targeted version of GFP (*white*). Genotype: *w* ^*118*^ *; Gal4* ^*21–7*^, *UAS-mCD8-GFP/UAS-Red-Stinger*. Scale bar is 100 μm. **b** Representative flow plots of *D. melanogaster* neurons. Plots show three progressive gates to identify RFP^+^ neurons, followed by two additional re-sorts with the same gates to assess purity. Compensated fluorescent values are shown. **c** Box plots generated in FastQC show average (*blue lines*) and median (*red lines*) quality scores across all read positions for each of the four independent replicate samples

Previous work has shown that quality-based trimming of RNA-Seq data can lead to greatly increased mappability of reads (*i.e.*, percentage of input reads that can be successfully aligned to a genome) [6]. However, this increased mappability of reads remaining after trimming comes at the expense of a dramatic reduction in the absolute number of aligned reads, as a consequence of some reads failing to pass minimum quality criteria during trimming. We predicted that this loss of information would impact analyses downstream of alignment; in particular, gene expression estimation. To assess this, we first generated RNA-Seq data from multi-dendritic (md) sensory neurons from *D. melanogaster* larvae, which had not yet been transcriptionally characterized by RNA-Seq despite their frequent use as a model system for neuronal development [11]. This approach was selected over those based on cells grown in culture to maximize physiological relevance. In this regard, the influence of trimming on expression measurement is particularly relevant to approaches using RNA-Seq for systematic identification of cell type in the nervous system [12, 13]. Neurons were sorted to high purity using two consecutive rounds of flow cytometry (Fig. 1a, b) and four samples comprised of 100 cells each were processed by SMART-Seq and sequenced on a HiSeq 2500. Each sample comprised at least seven million unpaired 51 base reads and was of high overall quality (Fig. 1c).

**Fig. 2:**
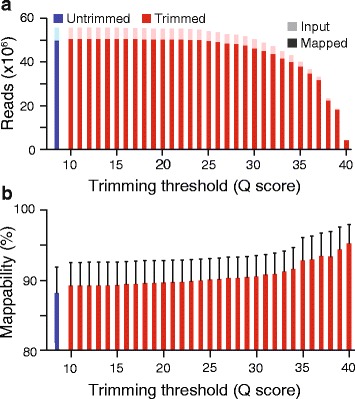
Influence of quality-based trimming on mappability. **a** The total number of input reads (*light bars*) and reads aligned to the transcriptome (*dark bars*) from four RNA-Seq data sets trimmed at a range of quality scores with SolexaQA. **b** The mappability, or number of aligned reads per total input reads per sample. Input reads shorter than 12 bases were not included in the calculation, as these are discarded by TopHat2 prior to alignment. Error bars represent standard deviations

To assess whether trimming improved mappability of our samples, as has been reported elsewhere [6], we trimmed our sensory neuron data sets with three different trimming algorithms and determined mappability. First, we evaluated SolexaQA, a sliding window trimmer that offers a balanced tradeoff between mappability and the number of mapped reads [6, 14]. We also evaluated Trimmomatic, which was shown to achieve high mappability with less aggressive trimming [6, 15], and ConDeTri, which demonstrated high mappability when used aggressively [6, 16]. We varied the quality score threshold from 10, corresponding to a 1 in 10 chance of an incorrect base, up to 40, corresponding to a 1 in 10,000 chance of an incorrect base – the highest confidence score assigned in Illumina sequencing data. After trimming, data were aligned to the annotated *D. melanogaster* transcriptome using TopHat2 [17]. As previously shown with another high quality RNA-Seq data set [6], mappability increased with increasing quality requirements, but the absolute number of aligned reads decreased (Fig. 2, SolexaQA; Additional file 1, Trimmomatic and ConDeTri). Thus, the impact of trimming on the mappability of the high quality reads generated from the small cell numbers employed in our study was similar to that observed from samples generated from abundant input RNA [6].

### Junction spanning reads decrease disproportionately following trimming

**Fig. 3:**
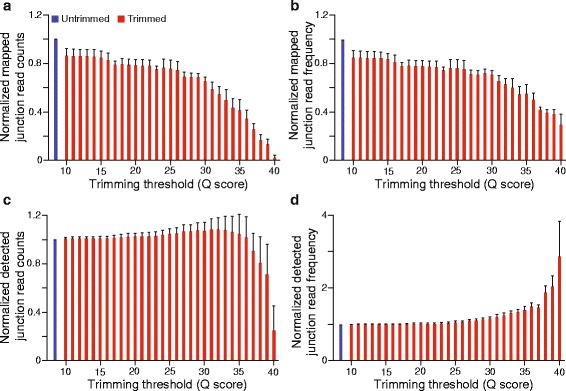
Influence of trimming on junction alignment and detection. **a** The average number of reads aligned to junctions per sample at a range of SolexaQA quality scores. **b** The average frequency of reads aligned to junctions (number of reads aligned to junctions per total reads aligned). **c** The average number of junctions detected per sample. **d** The average frequency of junction detection (number of junctions detected per total reads mapped). For all panels, data were normalized to the untrimmed value on a per sample basis. Error bars represent standard deviations

Although trimming increases overall mappability, it can also substantially shorten many reads, depending on the aggressiveness of the trimming parameters. We reasoned that this reduction in information content might introduce one or more forms of bias during read alignment. In particular, we predicted that there would be a disproportionate bias against reads aligning to exon-exon junctions, since alignment to such sites requires sequences long enough to span both the splice donor and acceptor sides of the junction. TopHat2 requires that reads, either singly or in combination with other reads, align for at least eight contiguous bases with no mismatches on both sides of a junction for initial junction detection, though subsequent reads may span a shorter distance and will still map to the junction [17, 18]. This is in contrast to aligning to non-junction locations, which minimally requires twelve contiguous bases with no more than one mismatch. As predicted, we observe that trimming disproportionately decreases the proportion of reads mapped to exon-exon junctions. The frequency of reads aligned to junctions, as a function of the total number of reads aligned, decreases as trimming quality score threshold increases, from 8.5 % (4.27 million reads aligned to junctions per 50.34 million total reads aligned in all samples combined) without trimming to 3.0 % (0.14 million reads per 4.54 million total reads) at Q40 (Fig. 3a, b). Interestingly, this is not the case with the frequency at which junctions are *detected*, as the number of junctions detected per reads aligned increases with increasing quality score stringency, from 1.5 junctions detected per thousand reads mapped without trimming (74 thousand junctions detected) to 4.3 junctions detected per thousand reads mapped at Q40 (20 thousand junctions detected) (Fig. 3c, d). Although the reason for this is unclear, we speculate that at the read coverage depth in our data, our ability to detect junctions is not constrained by coverage even after trimming, resulting in the increased frequency of junction detection largely being driven by the decrease in the total number of aligned reads.

### Bias in expression levels estimated from untrimmed and trimmed reads

We predicted that the decreased frequency of reads aligning to junctions would change estimates of isoform expression levels, since accurate alignment of reads to junctions contributes to the assignment of reads to specific isoforms [19]. Such bias would be expected to manifest as significantly different expression between trimmed and untrimmed samples, which we tested using Cuffdiff2 [20]. We note that throughout this work we refer to bias in the sense that gene expression is different between the groups, but with limited *a priori* knowledge of whether the gene expression estimates based on untrimmed or trimmed reads are more accurately reflective of the true expression levels (discussed in more detail below).

**Fig. 4:**
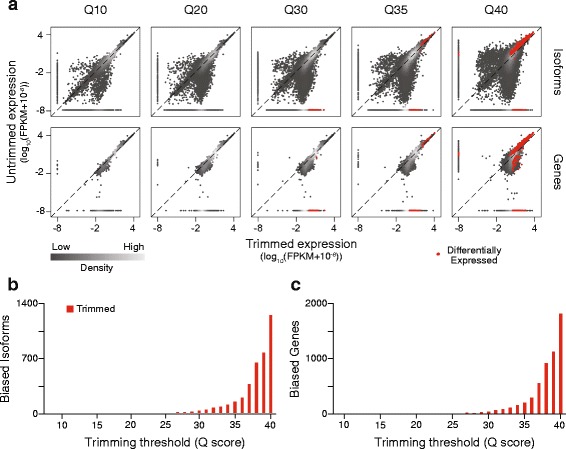
Isoform and gene expression levels after trimming. **a** Comparison of the expression estimates of isoforms and genes between increasing SolexaQA trimming thresholds (Q scores) and the untrimmed data set. Red dots represent statistically significant differential expression between data sets. **b** The number of significantly biased isoforms and (**c**) genes at a range of quality scores

As predicted, the expression of many isoforms was significantly altered by quality score trimming, with hundreds of differentially expressed isoforms detected with aggressive trimming (Fig. 4a, b). This finding holds even if novel junction discovery, the default behavior of TopHat2 mapping, is disabled (Additional file 2), since only the annotated transcriptome and junctions were used for modeling by Cuffdiff2. Because Cuffdiff2 estimates gene-level expression as the sum of the expression of all individual isoforms [19], we further predicted that in addition to isoforms, genes would exhibit expression bias following trimming. As expected, we observed a progressively increasing number of significant differentially expressed genes between our untrimmed data set and trimmed data sets with increasingly aggressive quality filtering (Fig. 4a, c). At the most stringent quality score, Q40, Cuffdiff2 identified 1829 genes, representing 10.5 % of all annotated genes, biased towards higher expression in either the untrimmed or trimmed data set, suggesting that trimming can have a substantial effect on the apparent composition of a sample.

Although the junction-alignment bias described above might play a role in these differential expression estimates, other factors must contribute as well since junction bias alone was insufficient to explain all of the observed bias. For example, we found that loss of junction reads did not uni-directionally decrease expression estimates. Instead, bias toward higher expression in untrimmed data was detected for some isoforms, but toward higher expression in trimmed data for others, including comparisons in which the number of junctions was held constant. Low expression level was also not a primary factor driving significance—no significant genes or isoforms exhibited expression values, measured as fragments per kilobase of transcript per million mapped reads (FPKM), of less than one in both the untrimmed and trimmed data sets (Fig. 4). Thus, it is likely that trimming introduces or corrects multiple sources of bias in gene expression estimation, relative to untrimmed reads, and that filtering based on expression level would not provide a means by which to eliminate this bias.

### Short trimmed reads are the predominant source of bias

**Fig. 5:**
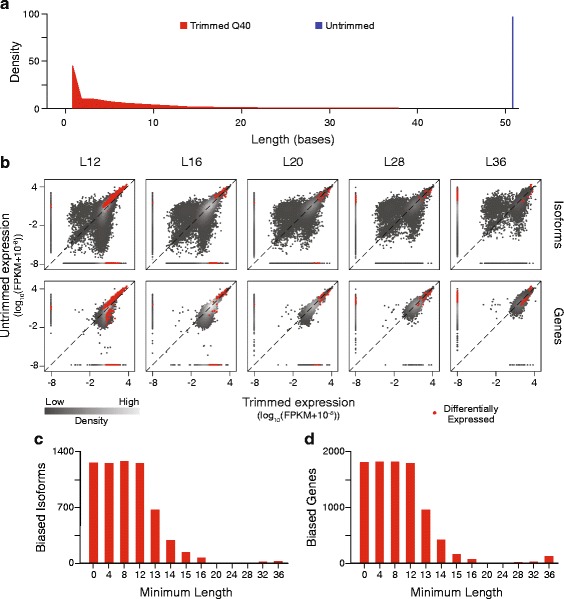
Isoform and gene expression levels after length-filtering. **a** The distribution of input read lengths before (*blue*) and after (*red*) trimming. **b** Comparison of the expression estimates of isoforms and genes between the untrimmed data set and the trimmed data sets with minimum read lengths required. L# specifies the minimum read length, in bases, required for inclusion. Red dots represent statistically significant differential expression between data sets. **c** The number of significantly biased isoforms and (**d**) genes at a range of minimum read lengths

Since bias resulting from differential alignment of junction-spanning reads could not fully account for the observed differences in expression estimated from untrimmed and trimmed reads, we next hypothesized that read length might contribute to the observed bias through other mechanisms. In addition to removing reads of very low quality in their entirety, trimming also shortens reads of mixed quality to preserve only high quality bases. Thus, the trimmed data sets have a distribution of read lengths as compared to the uniform read length in the untrimmed data set (Fig. 5a, Additional file 3). We predicted that shorter reads would align to more locations than longer reads, and that this promiscuity in mapping would drive some of the observed differential expression estimates. To evaluate this, we removed all reads below a fixed length in the most heavily trimmed SolexaQA data set, Q40-trimmed, and compared gene expression between these data and untrimmed reads. Minimum length requirements below 12 bases had no effect on the number of differentially expressed genes or isoforms identified by Cuffdiff2, which was expected since such reads fall below the default threshold for reads that TopHat2 attempts to align. However, following length filtering using longer thresholds, much of the bias both in isoform and gene expression between untrimmed and trimmed samples was eliminated (Fig. 5b-d). At the highest quality score, Q40, the number of significantly biased genes was reduced from 1829 to 150 and the number of significantly biased isoforms was reduced from 1269 to 41 when the minimum read length was increased from 1 to 36. Increasing stringency beyond a minimum of length of 36 was not attempted because few Q40-trimmed reads exceeded this length.

The impact of short reads on trimming-induced bias was corroborated by results from trimming with Trimmomatic and ConDeTri. Rather than searching sequencing reads for the longest run of bases over a given quality, both of these trimmers search from the end of reads, such that if a stretch of high quality is encountered near one of the ends, only the bases outside of that run will be truncated. One consequence of this approach to trimming is that the retained reads are considerably longer, with very few short reads retained as compared with SolexaQA (see Additional file 3). Consistent with the hypothesis that read length drives bias, even fairly aggressive application of these trimmers results in considerably less bias than trimming with SolexaQA, with a maximum of 9 biased genes with Trimmomatic (q = 30) and 28 biased genes with ConDeTri (hq = 39, lq = 34). Thus, short reads generated upon trimming are an important driver of bias in gene expression estimates, but this can be partially offset by imposing stringent minimum length filters.

Finally, we note that the long reads that remain after both stringent quality-based trimming and length filtering can be mapped with high accuracy; over 97 % of 36-mers present in the *D. melanogaster* genome are unique. Given that bias is minimized between the full, untrimmed data set and this aggressively trimmed and length filtered high confidence data set, this suggests that the full, untrimmed data set generates a more faithful representation of true gene expression estimates than those derived from aggressively trimmed data containing short reads.

### Additional factors contribute to gene expression bias

Although imposing read length requirements counteracted bias introduced by trimming, notable differences remained between the untrimmed and the processed data, and we next sought to identify additional drivers that could account for the residual bias. We divided the genes and isoforms differentially expressed at Q40 without length filtering into two groups—correctable and resistant—according to whether or not expression bias could be corrected by length filtering (minimum length = 36), as assessed using Cuffdiff2.

**Fig. 6:**
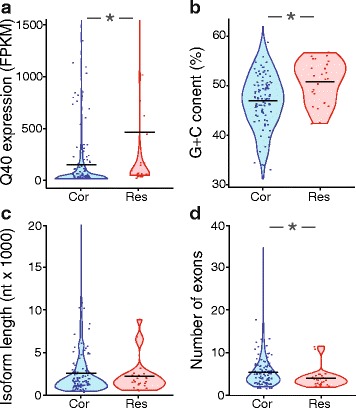
Correlations of gene and isoform properties with length filtering-resistant bias. **a** The distribution of gene expression levels (FPKM) for length filtering-correctable and -resistant genes, after SolexaQA trimming at Q = 40 and prior to length filtering. **b** The fractional GC content of correctable and resistant isoforms. **c** The distribution of transcript lengths for correctable and resistant isoforms. **d** The distribution of the number of exons per isoform for correctable and resistant isoforms. *, *p* < 0.05 following Benjamini-Hochberg adjustment. Bars represent the mean. For clarity, not all data points are depicted. Cor, correctable. Res, resistant

We assessed five parameters related to read alignment and transcript structure of the biased genes and isoforms. We hypothesized that poorly expressed genes would be more strongly impacted by promiscuous alignment of short reads than highly expressed genes, due to the proportion of inappropriately aligning reads being higher for poorly expressed genes. Consistent with this prediction, the expression levels of resistant genes and correctable genes differed prior to length filtering, with the resistant genes exhibiting a median expression of 56 FPKM, as compared with a median expression of 28 FPKM among the biased genes corrected by length filtering (*p* < 0.05, Mann–Whitney test) (Fig. 6a).

Because short reads are more likely to map to multiple locations in the genome (referred to as “multi-hits” for consistency with TopHat2 nomenclature), we next investigated how this property is associated with the observed biases. Before length filtering, multi-hit reads mapped to over 99 % of detected genes, indicating that expression estimates were broadly influenced by short reads aligning to multiple locations. However, this was not the case after imposing a minimum read length requirement of 36 bases: after filtering, 10 % of genes resistant to bias-correction, but only 1.8 % of correctable genes, contained any multi-hit reads (*p* < 0.05, Poisson test). Thus, mapping of non-unique short reads is rampant in aggressively trimmed data, and may continue to contribute a small portion of the residual bias even after length filtering. To more directly assess the role of multi-hits in differential expression following trimming, we repeated differential expression analysis using only uniquely mapping reads. Eliminating multi-hit reads greatly reduced the number of differentially expressed genes and isoforms after trimming at Q40 to 75 and 61, respectively (Additional file 4). However, as would be predicted based on the low percentage of non-unique reads present after length filtering, the effect on differential expression following length filtering was minimal (see Additional file 4), suggesting that multi-hits are not the primary driver of the residual bias after length filtering, and that additional factors may play a role. Although these data indicate that gene expression estimation from trimmed reads is stabilized by excluding multi-hits, others have found that allowing multi-hits increases the accuracy of expression estimates from 36-base RNA-Seq reads [21]. Thus, exclusion of all multi-hits could introduce bias as well; whether this bias or that associated with promiscuous alignment of short reads is more tolerable will need to be evaluated on a case-by-case basis.

The ability of short reads to align to multiple locations might be influenced by the intrinsic sequence content of a given gene or isoform. Specifically, we predicted that bias-correctable genes might exhibit lower sequence complexity, which would result in higher rates of multi-hit mapping, but that could be corrected by length filtering. To examine sequence complexity, we assessed entropy of isoform sequences in the two groups using Markov models for 1 to 6 base pair oligonucleotides [22]. Two of the six measures of complexity were significantly different between the correctable and resistant groups, with the correctable group exhibiting lower complexity in both cases as predicted (Additional file 5). However, we also noted that length filtering-resistant isoforms exhibited significantly higher GC content (Fig. 6b), and that both of the significant complexity measures were also significantly correlated with GC content. This observation suggested that GC content, rather than complexity per se, might be the primary underlying factor driving resistance to correction by length filtering. Notably, genes with high GC content exhibit disproportionately high expression values in RNA-Seq studies [23], which is also consistent with our observation that FPKM is associated with resistance to bias-correction (Fig. 6a). In anticipation of this potential bias, Cuffdiff2 was run with the optional fragment bias correction protocol [19] enabled; however, as evidenced by the above findings, some GC content bias remained.

We next evaluated structural properties of transcript isoforms—specifically, isoform length and number of exons—as a source of resistance to bias-correction through length filtering. The distributions of transcript lengths were not different between the two groups (*p* > 0.05, Mann–Whitney test) (Fig. 6c). In contrast, the number of exons, and therefore also the number of junctions, was higher in the correctable group (4.7 exons per isoform) as compared with the resistant group (3.2 exons per isoform) (Fig. 6d) (*p* < 0.05, Mann–Whitney test). In addition, both the frequency of junction detection and frequency of reads mapped to junctions increased with increasingly stringent length filtering (Additional file 6). Together, these data suggest that length filtering of quality-filtered data improves detection of exon-exon junctions in addition to reducing spurious multi-hit alignments.

### Trimming-induced differential expression is manifest in diverse analysis pipelines

Although TopHat2 and Cufflinks2 are widely used for analyzing RNA-Seq data, alternative tools have been gaining broad acceptance. Different tools vary in their underlying assumptions about read distribution and in their approach to handling non-uniquely mapping reads; therefore, we next examined whether the trimming-induced biases we identified are generalizable to other pipelines. Most tools assess differential expression based on gene-level counts, without discrimination of isoforms; thus, we focused our analysis on differential gene expression. We implemented four additional pipelines using the read aligners STAR [24] and RSEM [25] in combination with the differential analysis tools DESeq2 [26] and EdgeR [27]. Consistent with our TopHat2/Cufflinks2 results, significantly differentially expressed genes were detected with each additional pipeline following trimming with stringent quality parameters (SolexaQA with Q = 40), albeit fewer than our original analysis identified, and these largely disappeared when a length filter was imposed (Table 1). The differences in the number of differentially expressed genes between analysis tools may be due to inherent differences in how liberal or conservative the programs are in calling significant differences, as previously reported [28, 29]. Despite differences in the scale of the effect, all of these tools indicated that trimming affects gene expression estimates in this *D. melanogaster* RNA-Seq data set.

**Table 1:** Differentially expressed genes detected by multiple analysis pipelines

Mapping tool	DE tool	DE Genes, Q40	DE Genes, Q40 L36
TopHat2	Cuffdiff2	1829	150
TopHat2/HTSeq	DESeq	289	2
STAR	DESeq	812	2
RSEM/STAR	DESeq	79	53
STAR	EdgeR	321	0

The number of significantly differentially expressed genes detected, using 5 different analysis pipelines, when comparing the untrimmed data set to the same data set trimmed with SolexaQA, using a quality score of 40 (Q40), or with a quality score of 40 and a minimum length requirement of 36 bases (Q40 L36). DE Tool, differential expression tool. DE Genes, differentially expressed genes

### Trimming-induced differential expression is manifest in diverse RNA-Seq data sets

**Fig. 7:**
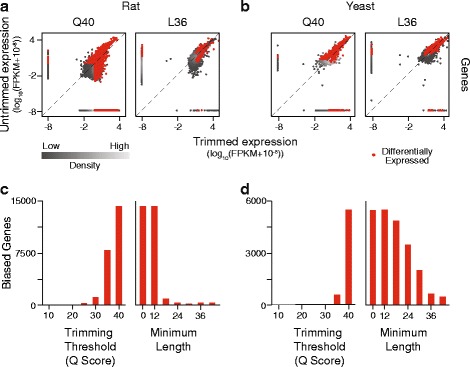
Differential gene expression following trimming in two additional data sets. **a** Comparison of the gene expression estimates from an RNA-Seq data set derived from rat samples, between SolexaQA trimmed data sets and the untrimmed data set. Q# specifies the quality threshold imposed. L# specifies the minimum read length, in bases, required for inclusion after Q40 trimming. Red dots represent statistically significant differential expression between data sets. **b** Comparison of the gene expression estimates from an RNA-Seq data set derived from yeast samples. **c** The number of significantly biased genes at a range of quality scores and lengths, rat data sets. **d** The number of significantly biased genes at a range of quality scores and lengths, yeast data sets

We next assessed whether the effects of trimming found in the *D. melanogaster* RNA-Seq data set were observed with other independently generated RNA-Seq data. For these analyses, we chose data sets derived from different organisms (rat livers [30], yeast cultures [31]) and generated in different labs using different library preparation and sequencing protocols. These additional data sets were comprised of samples with paired 101 base reads; thus, we anticipated that the negative effects of trimming would be less severe since longer reads are less likely to map to multiple locations, and paired reads must map concordantly. Instead, we found that trimming had a more pronounced effect on these data than on our original data (Fig. 7). Using the SolexaQA/TopHat2/Cuffdiff2 pipeline, we found that 54 % of genes (14,470 of 26,689 total) in the rat sample and 78 % of genes (5552 of 7126 total) in the yeast sample were significantly altered in their expression when the most aggressive trimming, Q40, was applied. As in the *D. melanogaster* data set, imposing a minimum length filter of 36 bases substantially reduced the number of differentially expressed genes, down to 2 % (rat) and 10 % (yeast) of all genes. We note that smaller fold changes between the trimmed and untrimmed samples were called as significantly different (visualized as points close to the identity line in Fig. 7a, b) in these two data sets than in the original data set, which might be due to lower variance between replicate samples and/or increased accuracy in alignments due to the use of paired reads. Thus, we expect that quality-based trimming will alter gene and isoform level expression estimates across RNA-Seq data sets, though the extent to which estimates change will depend on characteristics specific to each data set.

### Aggressive trimming decreases concordance with microarray expression estimates

Given that trimming causes substantial changes in gene expression estimates across multiple RNA-Seq data sets, we next investigated whether trimming improved or reduced the accuracy of expression estimates. As a biological standard for gene expression, we used the rat and yeast data sets described above, in which the same RNA libraries were subjected to genome-wide gene expression analysis both by RNA-Seq and by hybridization to microarrays [30, 31]. Specifically, we reasoned that if trimming reduced the accuracy of RNA-Seq based expression estimates, we should observe decreased concordance between the RNA-Seq and microarray expression values in trimmed RNA-Seq data sets. This is precisely what we observed (Table 2). In the two independent RNA-Seq/microarray data sets, expression estimates from untrimmed RNA-Seq data were most highly correlated with microarray expression estimates, though even moderately aggressive trimming, up to Q30, minimally reduced these correlations. By contrast, aggressive trimming led to substantially reduced correlations with microarray data. Length filtering slightly improved the correlations with microarray estimates for the heavily trimmed rat data; however, length filtering of the yeast data further decreased correlations, suggesting that this additional filtering may not universally counteract trimming-induced bias. Thus, by validation with an independent technique, we conclude that no or low trimming thresholds are most likely to result in the highest accuracy for RNA-Seq based expression estimates.

**Table 2:** Correlations between RNA-Seq gene expression estimates and microarray intensities

Data set	Untrimmed	Q10	Q20	Q30	Q40	Q40 L36
Rat	0.855	0.853	0.851	0.848	0.744	0.751
Yeast	0.891	0.891	0.889	0.887	0.863	0.785

Values represent correlation coefficients between gene expression values determined by microarray data sets and RNA-Seq data sets that were trimmed with SolexaQA with quality scores as indicated, followed by mapping and modeling with TopHat2 and Cuffdiff2

## Conclusions

The data we present here provide evidence that aggressive quality-based trimming can strongly influence estimation of gene and isoform expression levels, which subsequently impacts identification of differentially expressed genes. A considerable source of the observed differences can be attributed to the alignment of shorter reads that result from trimming. Imposing minimum read length requirements reverts gene expression estimates to values closer to estimates produced from untrimmed reads, suggesting that untrimmed or trimmed, length-filtered reads—the latter of which likely represent the highest quality reads within a data set—may most accurately reflect the actual library composition.

Because different experiments have different goals, individual researchers must determine whether or not trimming will be beneficial for their particular application. For example, in genome sequencing or for RNA-Seq experiments where extremely large numbers of reads are available, modest trimming may provide benefits. Further, in data sets with low average base calling quality, or in library preparation protocols that are susceptible to adapter contamination, trimming may allow the recovery of reads which would otherwise be detrimental to expression estimation. Both of these attributes were more common in early RNA-Seq studies, so trimming may be particularly useful when re-analyzing such data. One potential improvement may be to use longer sequencing reads, such as 100 or 150 bases, so that longer reads remain after trimming low quality bases from either end, though our results demonstrate that this alone will not prevent the introduction of trimming-induced expression changes. However, we re-iterate previously voiced concerns [7, 8] that mappability should not be used as the sole criterion for performance. Furthermore, our results suggest that aggressive trimming adversely affects the accuracy of expression estimates. Therefore, if trimming is applied, extreme care should be used, and other measures such as length filtering should be considered in the pre-processing pipeline to minimize the introduction of unwanted bias.

## Methods

### Fly stocks

The following lines were used in this study: *Gal4*^*21–7*^ [32], *UAS-RedStinger* [33], *UAS-mCD8GFP* [34].

### Flow cytometry

Third instar larvae were filleted by microdissection in PBS. Internal organs and thoracic segments were removed, and the remaining body walls were digested in 500 μl 0.9 mg/ml (200 U/ml) collagenase in PBS for 18 min at 37 °C with mechanical agitation (1000 rpm on a 3 mm orbit diameter shaker, with trituration every 6 min). Debris was removed by filtering cell suspensions through a 70 μm nylon filter, and cells were isolated to high purity using two successive rounds of sorting on a FACSAria II (BD Biosciences, San Jose, CA). Four samples of 100 cells each were captured into 2 μl of SMARTer lysis mix (described below) and were immediately processed for RNA-Seq.

### RNA-Seq

Total RNA from lysed cells was converted to pre-amplified cDNA libraries using template-switching reverse transcription [35, 36] as implemented in the SMARTer Ultra-low input kit (Clontech, Mountain View, CA), but with modified procedures for low cell number analysis (Fluidigm, South San Francisco, CA). Pre-amplified cDNA libraries were diluted to 0.25 ng/ul. Fragmentation was performed enzymatically using a Nextera XT DNA kit (Illumina, San Diego, CA), and barcoded samples were multiplexed, pooled, purified using Agencourt AMPure XP beads (Beckman Coulter Genomics, Danvers, MA), and quality controlled on a Bioanalyzer 2100 using a high sensitivity dsDNA assay (Agilent Technologies, Santa Clara, CA). Quality-controlled libraries were sequenced as 51 base single end reads on a HiSeq 2500 running in high-output mode at the UCSF Center for Advanced Technology (San Francisco, CA). Reads were demultiplexed with CASAVA (Illumina), and read quality was assessed using FastQC (http://www.bioinformatics.babraham.ac.uk/projects/fastqc/). One library was sequenced twice in order to increase sequencing depth. In total, the four replicate samples were comprised of 7, 13, 14, and 21 million reads passing sequencing filters.

### Trimming with SolexaQA

Trimming was performed with SolexaQA version 3.1.2 [14], which scans for the longest contiguous run in the sequence with quality scores at or above the user-provided value. To perform filtering on read lengths, the lengthsort command was run following the initial trimming command. Example commands for these and all other tools can be found in Additional file 7.

### Trimming with Trimmommatic

Trimming was performed with Trimmomatic version 0.33 [15]. We used the quality filtering functionality of this tool with a sliding window, which scans through reads from the 5′ end, and removes following bases from the 3′ end once the average quality score within the window drops below a user-specified value.

### Trimming with ConDeTri

Trimming was performed with ConDeTri version 2.2 [16]. For each instance, both a high quality and a low quality score were provided as parameters; the low quality scores were held either five or ten below the high quality scores for all combinations tested. Briefly, ConDeTri removes bases from the 3′ end of reads that are below the high quality score. Once a base is encountered that surpasses the high quality score, bases are retained so long as the bases between the low quality score and high quality score, as a fraction of total bases, does not rise above a default threshold of 0.2. All bases distal to a base below the low quality threshold are discarded. Aside from the quality scores, the only other parameter that was altered from the defaults was the minimum length, which was removed rather than using the default value of 50 to accommodate the 51 base sequencing reads used in this study.

### Alignment to the transcriptome

After trimming, reads were aligned to the *D. melanogaster* genome, FlyBase genome release 6.04, to the *Rattus norvegicus* genome, Ensembl release 5.0, or to the *Saccharomyces cerevisiae* genome, Ensembl release R64-1-1. TopHat2 version 2.0.14 [17] and Bowtie2 version 2.2.3 [17, 37] were used for alignment using two threads, but otherwise with all default parameters. The aligned reads, alignment summary, and junction alignment files were used in further analysis. In addition to the above, several other alignment/expression estimation approaches were employed. In one case, gene-level counts from the TopHat2 output were determined using HTSeq version 0.6.0 [38]. All standard parameters were used in the gene counts mode for the aligner STAR version 2.4.2a [24]. RSEM version 1.2.22 [25] was used in combination with STAR version 2.4.2a [24].

### Gene expression analysis

Differential gene expression analysis was performed using Cuffdiff2 version 2.2.1 [20]. In each case, the three (yeast) or four (fly, rat) trimmed samples were compared to the three or four samples without any trimming. A reference transcriptome was provided, and as such any novel junctions detected by TopHat2 were not modeled. All other parameters were their default. The gene_exp.diff and isoform_exp.diff output files were used to determine the significantly differentially expressed genes and isoforms as well as expression values in both trimmed and untrimmed samples. For diverse pipeline analysis, differential gene expression analysis on counts data was performed using the R package DESeq2 version 1.10.0 [26] or the R package EdgeR version 3.13.4 [27].

### Gene and isoform parameter analysis

Gene and isoform parameters were generated from the Cuffdiff2 output (gene expression) and the FlyBase release 6.04 transcriptome (isoform length, number of exons per isoform). Significance in comparisons of these parameters was assessed using a Mann–Whitney *U* test. The number of genes to which multi-hit reads mapped was determined by identifying multi-hits using the TopHat2 output, followed by using these reads as input to Cufflinks version 2.2.1 [19]. All genes which showed non-zero expression from any of the four multi-hit samples were considered to be a target of multi-hit reads. Significance was assessed using a Poisson test. GC content and Markov entropy scores were calculated as previously described [22, 39] using a publicly available Perl package (https://github.com/caballero/SeqComplex.git). Significance was assessed using a two-tailed Student *t* test assuming unequal variances. An adjusted p-value of 0.05 after Benjamini-Hochberg correction was deemed significant.

### Correlations with microarray expression data

Microarray intensity values were retrieved from the NCBI Gene Expression Omnibus (GEO) with the R package GEOquery version 2.37 (https://github.com/seandavi/GEOquery). Probes were mapped to the same genome to which RNA-seq reads were aligned, and any probes mapping to more than one gene were discarded. The normalized intensity values were averaged across all samples and all probes mapping to each gene to calculate gene-level intensity values. Pearson’s correlations were used to measure the correlation between the average gene expression based on microarray intensity data and the estimated gene expression based on RNA-Seq data, after imposing a lower expression cutoff of 1 FPKM.

## Availability of supporting data

The fly data set generated in this article is available in the NCBI Sequence Read Archive (SRA) and in the Gene Expression Omnibus (GEO) under accession number GSE72884. The rat RNA-Seq data sets used were obtained from the SRA under accession numbers SRR1178065, SRR1178067, SRR1178068, and SRR1178069 and the corresponding microarray data sets were obtained from GEO under accession numbers GSM116428, GSM1161435, GSM1161439, and GSM1161443. The yeast RNA-Seq data sets used were obtained from the SRA under accession numbers SRR453569, SRR453570, and SRR453571, and the corresponding microarray data sets were obtained from GEO under accession numbers GSM923093, GSM923094, and GSM923095.

## Additional files

Additional file 1:
**Influence of trimming with Trimmomatic and ConDeTri on mappability.** (a) The total number of input reads (light bars) and reads aligned to the transcriptome (dark bars) from four RNA-Seq data sets trimmed at a range of quality scores with Trimmomatic. Q scores are 5 apart from 5 to 25, and every Q score from 25 to 34 is shown. No reads survived at or above a Q score of 35. (b) The mappability, or number of aligned reads per total input reads, per sample trimmed with Trimmomatic. (c) The total number of input reads (light bars) and reads aligned to the transcriptome (dark bars) from four RNA-Seq data sets trimmed with ConDeTri. The high quality (HQ) score for trimming is indicated under the first of each pair of bars, and a low quality (LQ) score five (red bars) or ten (orange bars) below was used. (d) The mappability per sample trimmed with ConDeTri. Input reads shorter than 12 bases were not included in the mappability calculations, as these are discarded by TopHat prior to alignment. Error bars represent standard deviations. (PDF 116 kb) Additional file 2:
**Influence of novel junction discovery on isoform and gene expression levels.** Comparison of the expression estimates of isoforms and genes between the SolexaQA Q40-trimmed and the untrimmed data set, after aligning reads to the transcriptome using TopHat2 with novel junction discovery disabled. Red dots represent statistically significant differential expression between data sets. (PDF 642 kb) Additional file 3:
**Distribution of read lengths after trimming.** Density plots show the distributions of read lengths at multiple Q scores following trimming with SolexaQA (a), Trimmomatic (b), and ConDeTri (c). (PDF 156 kb) Additional file 4:
**Influence of multi-hits on isoform and gene expression levels.** Comparison of the expression estimates of isoforms and genes between the SolexaQA Q40-trimmed without (a) or with (b) a minimum length requirement and the untrimmed data set, after aligning reads to the transcriptome using TopHat2 with multi-hits excluded. Red dots represent statistically significant differential expression between data sets. (PDF 1130 kb) Additional file 5:
**Relationship between length-filtering resistant bias and sequence complexity measures.** The distribution of complexity scores for length filtering-correctable and -resistant isoforms, assessed with a Markov model for entropy of oligonucleotides of length one (a), two (b), three (c), four (d), five (e), or six (f). *, *p* < 0.05 following Benjamini-Hochberg adjustment. Bars represent the mean. For clarity, not all data points are depicted. Cor, correctable. Res, resistant. (PDF 152 kb) Additional file 6:
**Influence of minimum length requirements on junction alignment and detection.** (a) The average number of reads aligned to junctions per sample with increasing minimum read length requirements after trimming with SolexaQA, Q = 40. (b) The average frequency of reads aligned to junctions (number of reads aligned to junctions per total reads aligned). (c) The average number of junctions detected per sample. (d) The average frequency of junction detection (number of junctions detected per total reads mapped). For all panels, data were normalized to the Q40 value with no minimum length filter, on a per sample basis. Error bars represent standard deviations. (PDF 114 kb) Additional file 7:
**Example commands used for analysis tools**. Table showing example commands for all analysis tools used. (PDF 57 kb)

## Abbreviations

FPKM: fragments per kilobase of transcript per million reads mapped
Q: Phred quality score
RNA-Seq: RNA sequencing
