# Supplementary figures and images for: Trimming of sequence reads alters RNA-Seq gene expression estimates
Source: BMC Bioinformatics. 2016 Feb 25;17:103. doi: 10.1186/s12859-016-0956-2 (PMC4766705; doi:10.1186/s12859-016-0956-2)

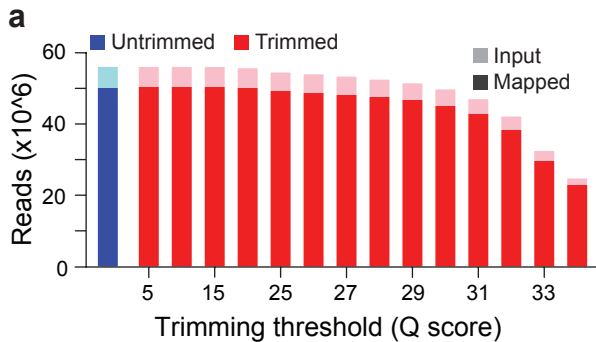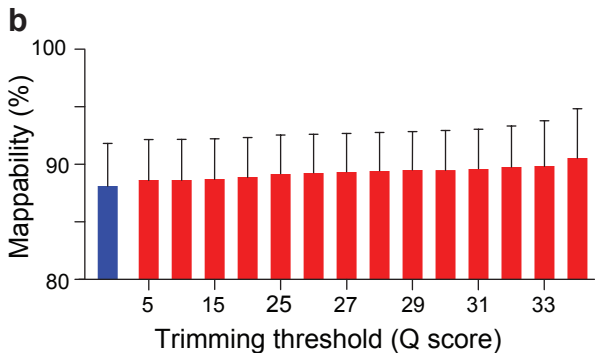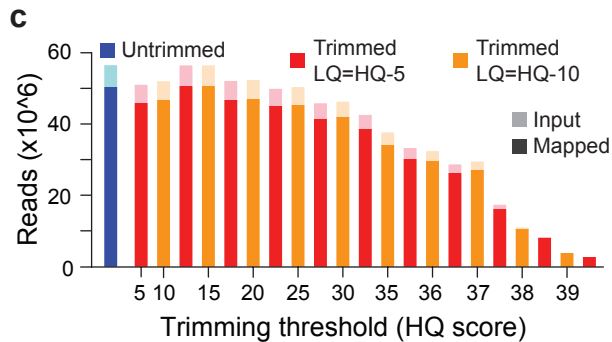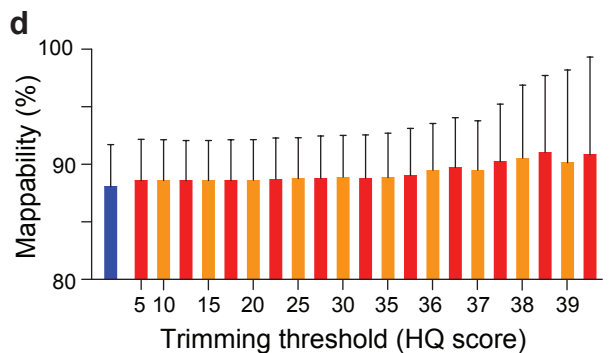

Supplement: Additional file 1: — Influence of trimming with Trimmomatic and ConDeTri on mappability. (a) The total number of input reads (light bars) and reads aligned to the transcriptome (dark bars) from four RNA-Seq data sets trimmed at a range of quality scores with Trimmomatic. Q scores are 5 apart from 5 to 25, and every Q score from 25 to 34 is shown. No reads survived at or above a Q score of 35. (b) The mappability, or number of aligned reads per total input reads, per sample trimmed with Trimmomatic. (c) The total number of input reads (light bars) and reads aligned to the transcriptome (dark bars) from four RNA-Seq data sets trimmed with ConDeTri. The high quality (HQ) score for trimming is indicated under the first of each pair of bars, and a low quality (LQ) score five (red bars) or ten (orange bars) below was used. (d) The mappability per sample trimmed with ConDeTri. Input reads shorter than 12 bases were not included in the mappability calculations, as these are discarded by TopHat prior to alignment. Error bars represent standard deviations. (PDF 116 kb) [file 12859_2016_956_MOESM1_ESM.pdf]

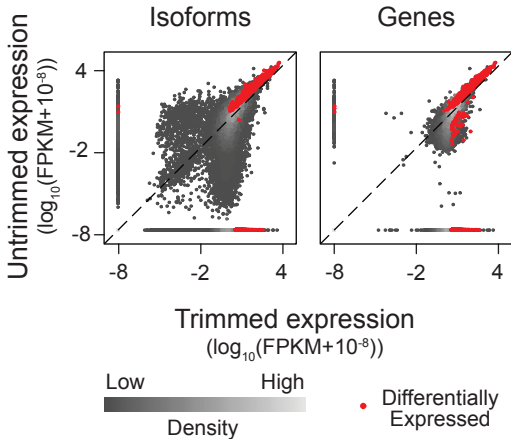

Supplement: Additional file 2: — Influence of novel junction discovery on isoform and gene expression levels. Comparison of the expression estimates of isoforms and genes between the SolexaQA Q40-trimmed and the untrimmed data set, after aligning reads to the transcriptome using TopHat2 with novel junction discovery disabled. Red dots represent statistically significant differential expression between data sets. (PDF 642 kb) [file 12859_2016_956_MOESM2_ESM.pdf]

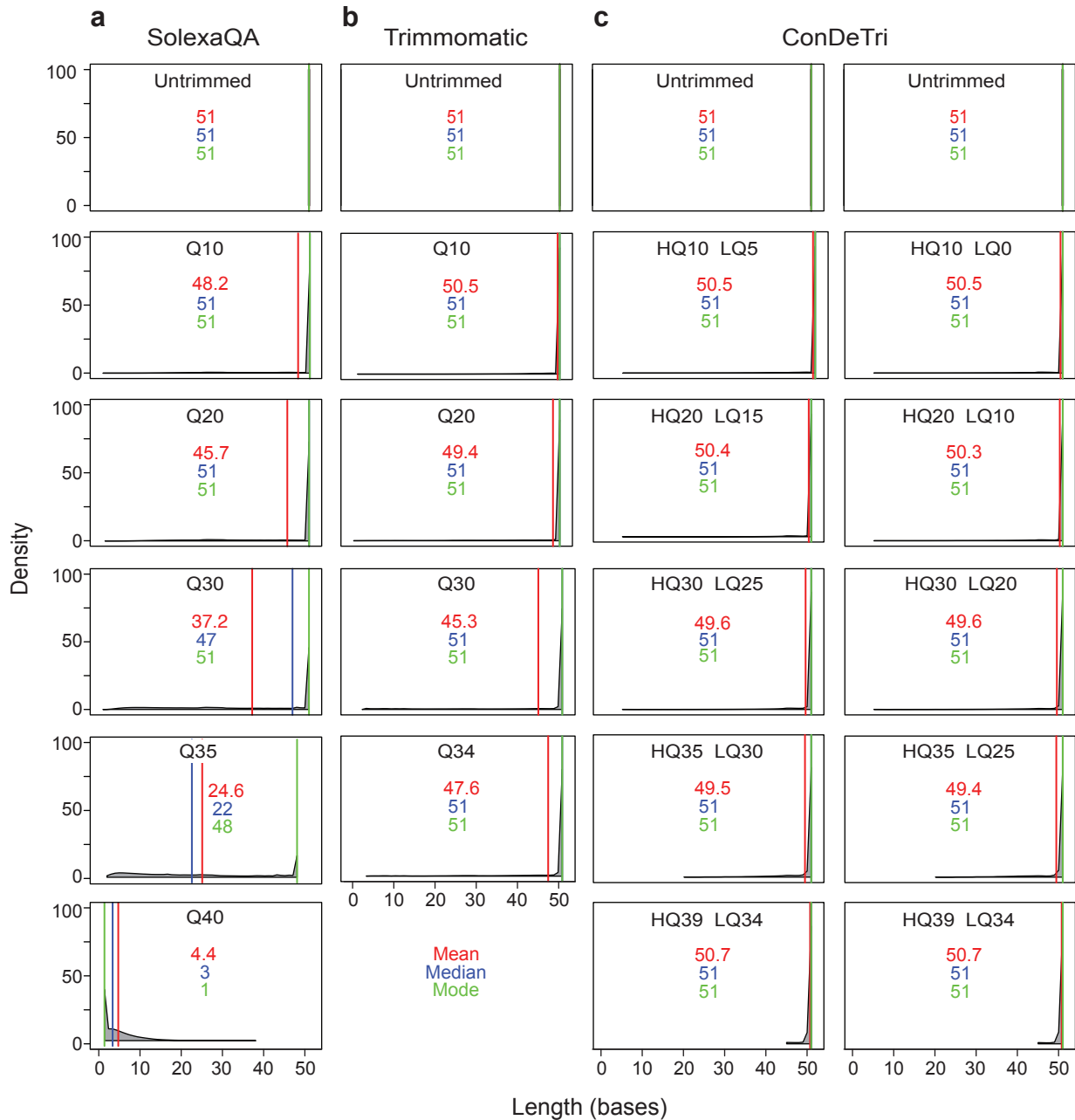

Supplement: Additional file 3: — Distribution of read lengths after trimming. Density plots show the distributions of read lengths at multiple Q scores following trimming with SolexaQA (a), Trimmomatic (b), and ConDeTri (c). (PDF 156 kb) [file 12859_2016_956_MOESM3_ESM.pdf]

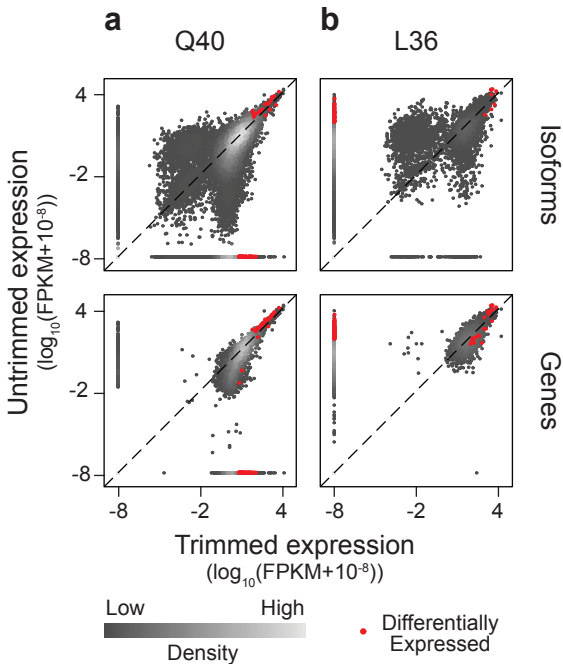

Supplement: Additional file 4: — Influence of multi-hits on isoform and gene expression levels. Comparison of the expression estimates of isoforms and genes between the SolexaQA Q40-trimmed without (a) or with (b) a minimum length requirement and the untrimmed data set, after aligning reads to the transcriptome using TopHat2 with multi-hits excluded. Red dots represent statistically significant differential expression between data sets. (PDF 1130 kb) [file 12859_2016_956_MOESM4_ESM.pdf]

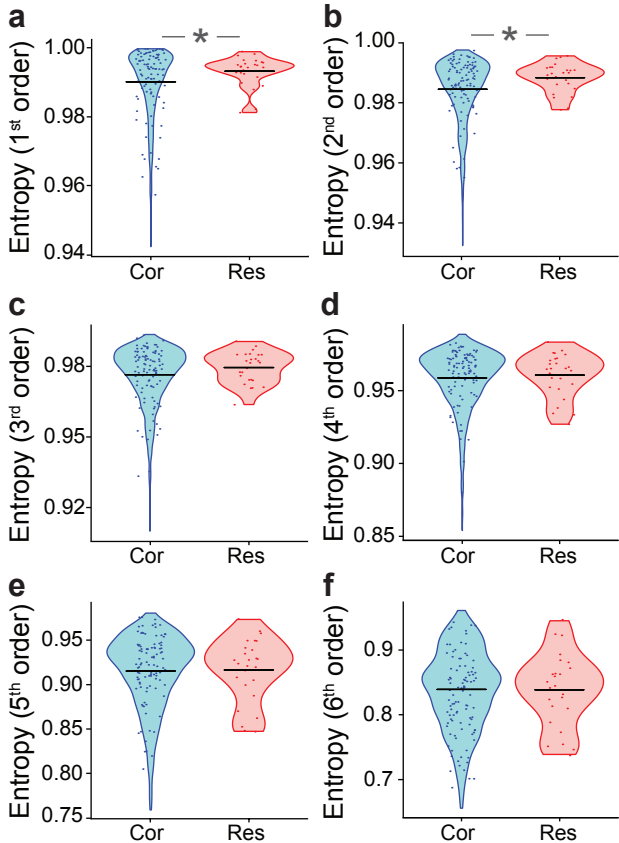

Supplement: Additional file 5: — Relationship between length-filtering resistant bias and sequence complexity measures. The distribution of complexity scores for length filtering-correctable and -resistant isoforms, assessed with a Markov model for entropy of oligonucleotides of length one (a), two (b), three (c), four (d), five (e), or six (f). *, p < 0.05 following Benjamini-Hochberg adjustment. Bars represent the mean. For clarity, not all data points are depicted. Cor, correctable. Res, resistant. (PDF 152 kb) [file 12859_2016_956_MOESM5_ESM.pdf]

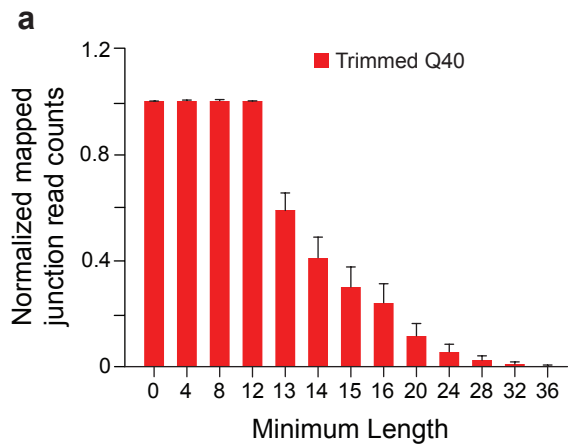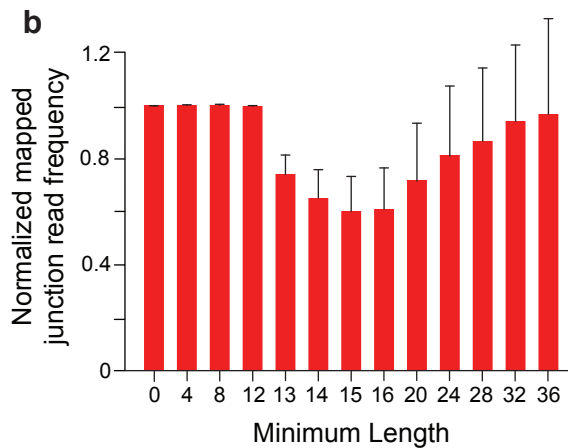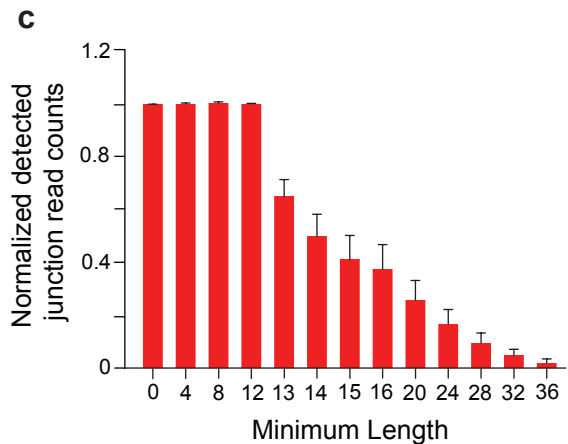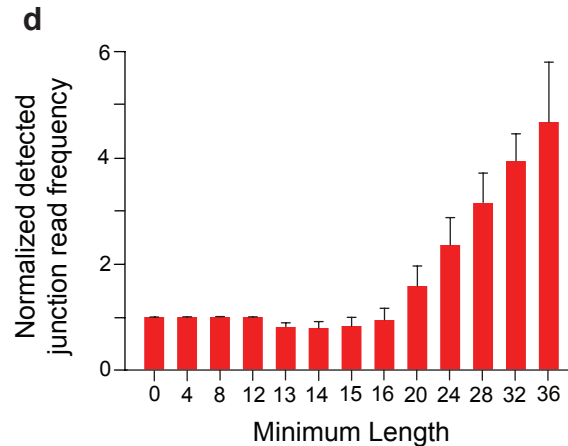

Supplement: Additional file 6: — Influence of minimum length requirements on junction alignment and detection. (a) The average number of reads aligned to junctions per sample with increasing minimum read length requirements after trimming with SolexaQA, Q = 40. (b) The average frequency of reads aligned to junctions (number of reads aligned to junctions per total reads aligned). (c) The average number of junctions detected per sample. (d) The average frequency of junction detection (number of junctions detected per total reads mapped). For all panels, data were normalized to the Q40 value with no minimum length filter, on a per sample basis. Error bars represent standard deviations. (PDF 114 kb) [file 12859_2016_956_MOESM6_ESM.pdf]
