# Supplementary material for: Trimming of sequence reads alters RNA-Seq gene expression estimates
Source: BMC Bioinformatics. 2016 Feb 25;17:103. doi: 10.1186/s12859-016-0956-2 (PMC4766705; doi:10.1186/s12859-016-0956-2)
Supplement: Additional file 7: — Example commands used for analysis tools. Table showing example commands for all analysis tools used. (PDF 57 kb) [file 12859_2016_956_MOESM7_ESM.pdf]

Additional File 7. Commands used for analysis tools.

Analysis Tool	Use	Example Command
SolexaQA	Trimming	SolexaQA++ dynamictrim -h 10 pns1.fastq
SolexaQA	Length filtering	SolexaQA++ lengthsort -l 36 pns1.fastq.trimmed
Trimmomatic	Trimming	java -jar trimmomatic-0.33.jar SE -threads 2 -trimlog pns1_trimlog -phred33 pns1.fastq pns1_trim.fastq SLIDINGWINDOW:4:10
ConDeTri	Trimming	condetri_v2.2.pl -fastq1=pns1.fastq -prefix=pns1_cdt -hq=10 -lq=5 -minlen=1 -sc=33
Tophat2	Mapping	tophat2 -p 2 --transcriptome-index=genes_all genome_all pns1.fastq.trimmed
Tophat2	Mapping, multi-hits excluded	tophat2 –p 2 –g 1 –M –x 1 --transcriptome-index=genes_all genome_all pns1.fastq.trimmed
Tophat2	Mapping, novel junctions disabled	tophat2 -p 2 --transcriptome-index=genes_all --no-novel-juncs genome_all pns1.fastq.trimmed
HTseq	Counting alignments	htseq-count –stranded=no accepted_hits.sam genes_all.gtf > accepted_hits.counts
STAR	Mapping	STAR --runThreadN 2 --genomeDir fb604genome/star/ --readFilesIn pns1.fastq.trimmed --quantMode GeneCounts
RSEM / STAR	Mapping	rsem-calculate-expression --star –p 2 pns1.fastq.trimmed fb604genome/rsem/genome_all pns1
Cuffdiff2	Differential expression	cuffdiff -b genome_all.fa -p 2 -L untrimmed,trimmed -u genes_all.gtf untrimmed/pns1/accepted_hits.bam,untrimmed/pns2/accepted_hits.bam,untrimmed/pns3/accepted_hits.bam,untrimmed/pns4/accepted_hits.bam trimmed/pns1/accepted_hits.bam,trimmed/pns2/accepted_hits.bam, trimmed/pns3/accepted_hits.bam,trimmed/pns4/accepted_hits.bam
DESeq2	Differential expression	R functions used were: DESeq, results
EdgeR	Differential expression	R functions used were: calcNormFactors, estimateCommonDisp, estimateTagwiseDisp, exactTest, and topTags
